# Supplementary material for: Multiple low dose therapy as an effective strategy to treat EGFR inhibitor-resistant NSCLC tumours
Source: Nat Commun. 2020 Jun 22;11:3157. doi: 10.1038/s41467-020-16952-9 (PMC7308397; doi:10.1038/s41467-020-16952-9)
Supplement: Supplementary file 1 — Supplementary Information [file 41467_2020_16952_MOESM1_ESM.pdf]

Supplementary Information

Multiple Low Dose therapy as an effective strategy to treat EGFR inhibitor-resistant NSCLC tumours

Fernandes Neto et al.

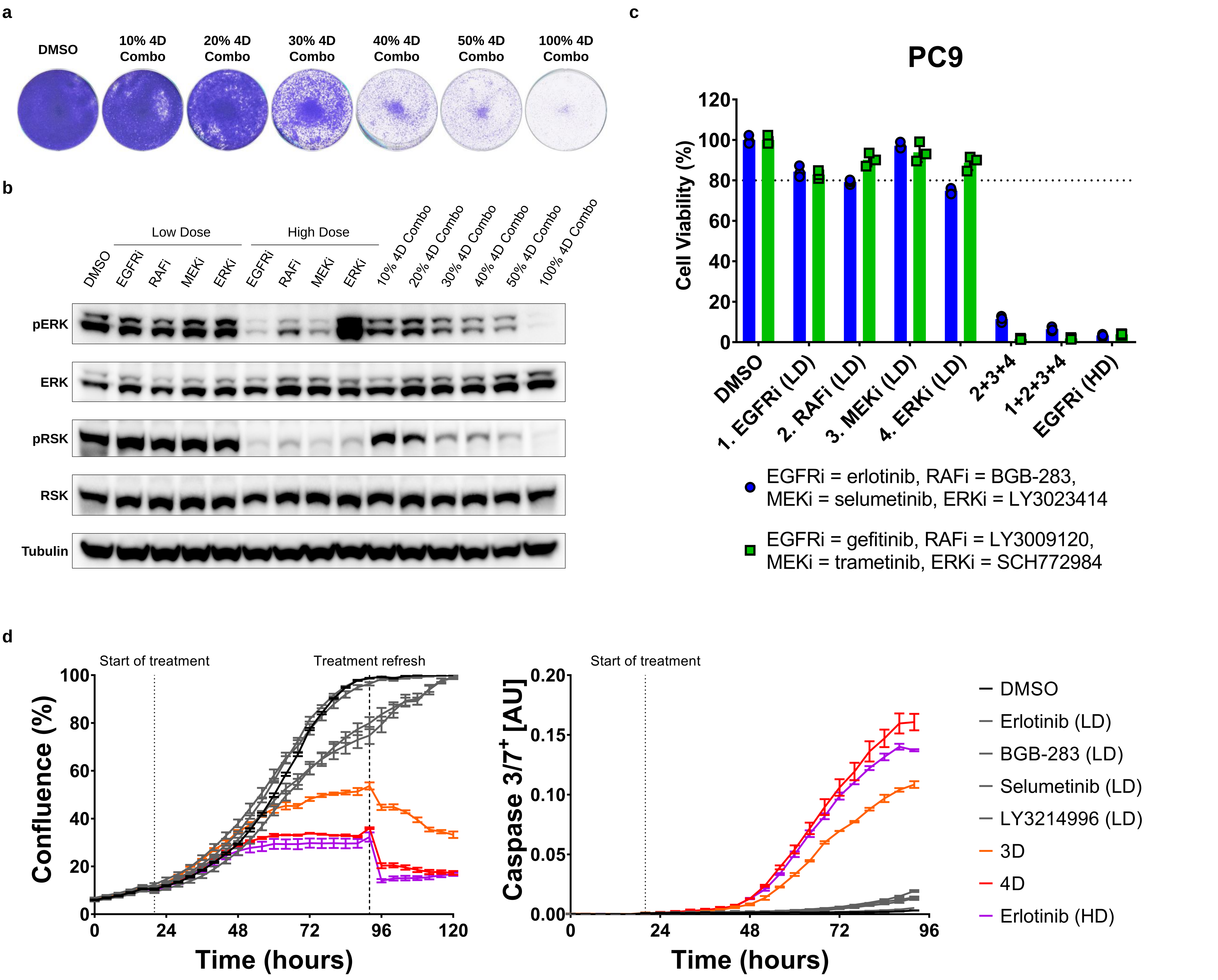

Supplementary Figure 1: A drug concentration threshold is necessary for the efficacy of MLD therapy, which is not drug-specific.
a, Dilution of 4D Combo results in incomplete inhibition of proliferation. PC9 cells were plated and incubated overnight to allow attachment to the plate. Cells were then treated with DMSO, with 4D Combo and with the indicated dilutions of 4D Combo. Cells were cultured for 7 days, after which plates were stained and scanned; A representative image from 3 biologically independent replicates is displayed. **b,** Dilution of 4D Combo results in incomplete MAPK pathway inhibition. PC9 cells were cultured with DMSO, with EGFR, RAF, MEK and ERK inhibitors both at low and at high doses, with 4D Combo and with different dilutions of 4D combo. Protein for western blotting was harvested after 24 hours of treatment. The level of pathway inhibition was measured by examining pERK and pRSK protein levels; Tubulin was used as loading control. **c, d,** MLD therapy efficacy is not drug-specific. PC9 cells were plated and incubated overnight to allow attachment to the plate; Cells were then treated with two different inhibitors for each of the nodes in the MAPK pathway (gefitinib or erlotinib as EGFRi, LY3009120 or BGB-283 as RAFi, Trametinib or selumetinib as MEK and SCH772984 or LY-3214996 as ERKi) as indicated. In **c** cell viability was measured using CellTiter-Blue® after 4 days of treatment. Standard deviation (SD) from 3 replicates is plotted. In **d** the confluence (left) and caspase 3/7 activation (right) over time was measured by the IncuCyte®; 3 days after the first treatment the drugs and media were refreshed. SEM from 3 replicates is plotted.

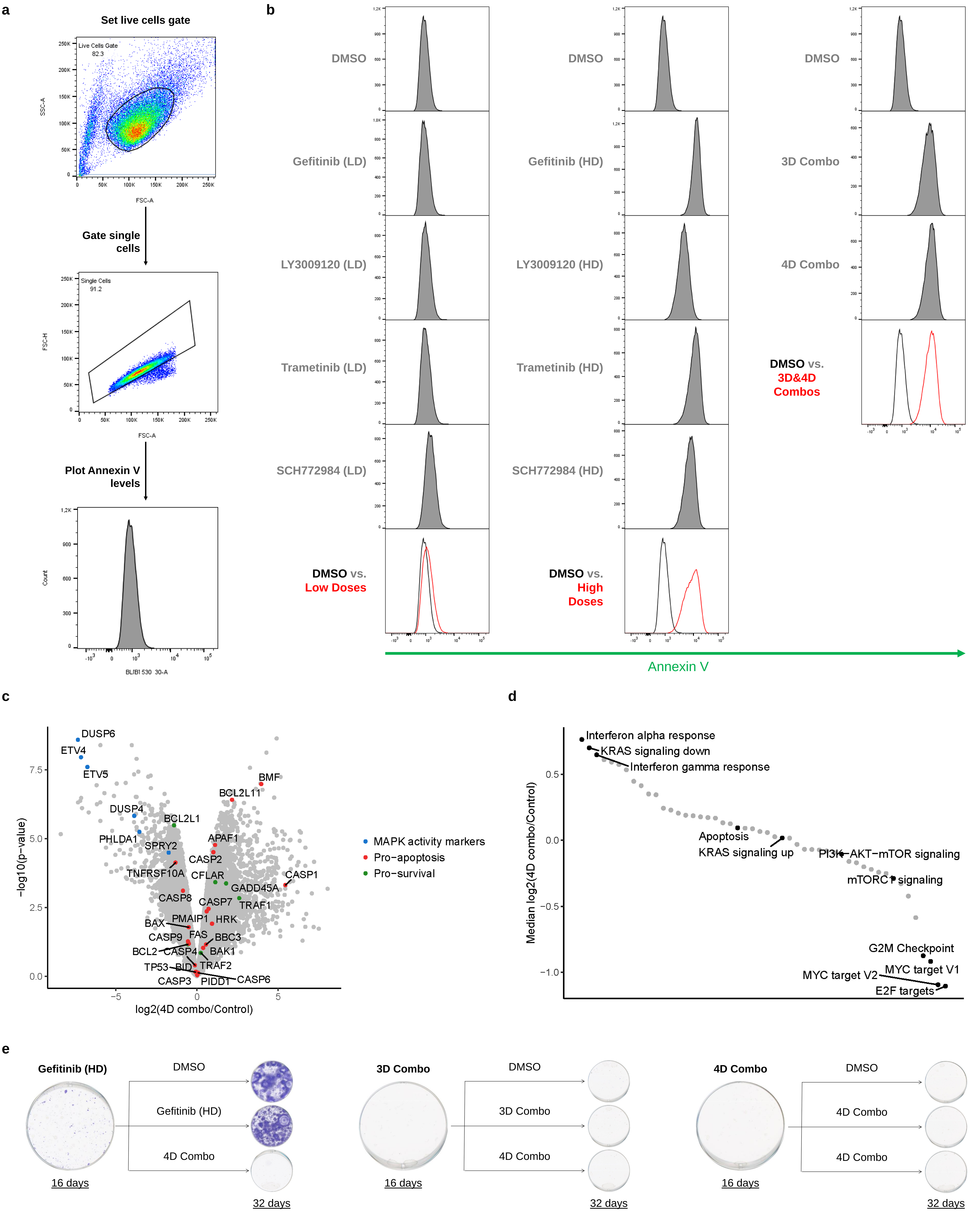

Supplementary Figure 2: MLD therapy induces apoptosis and prevents drug resistance.
a, Gating strategy used in **b**. Live cells were gated from all events; then single cells were gated from the live cells and, finally, Annexin V levels were plotted from the single cells. **b,** 3D and 4D Combos induce apoptosis at comparable levels as high doses of each inhibitor in PC9 cells. PC9 cells were stained with Annexin V-FITC Apoptosis Staining/Detection kit (ab14085) after 48 hours of drug treatment. The Annexin V levels were measured by flow cytometry (BD LSRFortessa) and analysed using FlowJo V10. **c, d,** Transcriptome analysis of PC9 cells treated with 4D combo. **c**, Volcano plot of differential gene expression analysis. **d**, Median log2-fold change of the MSigDB hallmark gene-sets, ranked from high to low. For **c** and **d** PC9 cells were treated with DMSO for 48 hours or with 4D combo for 48 or 72 hours. Experiments were performed in duplicates. Because the difference between 48 and 72 hour 4D combo treatment was comparable to the variability between replicates, the four MLD treated samples were considered replicates. Differential expression analysis was performed using the R-package limma [Ritchie et al, 2015] and the MSigDB hallmark gene-sets analysis was performed using version 6.2 of MSigDB [Liberzon et al, 2015]. **e,** MLD therapy prevents the acquisition of drug resistance in PC9 cells. PC9 cells were cultured with high dose of gefitinib (280 nM) and with 3D and 4D Combos (4 plates per condition). After 16 days in culture, one plate was fixed and stained. From the remaining three plates (per condition) one was switched to DMSO treatment, the other was switched to 4D Combo and the third one continued with the previous treatment. Sixteen days later (after 32 days of “treatment” in total) cells were fixed and stained and then plates were scanned.

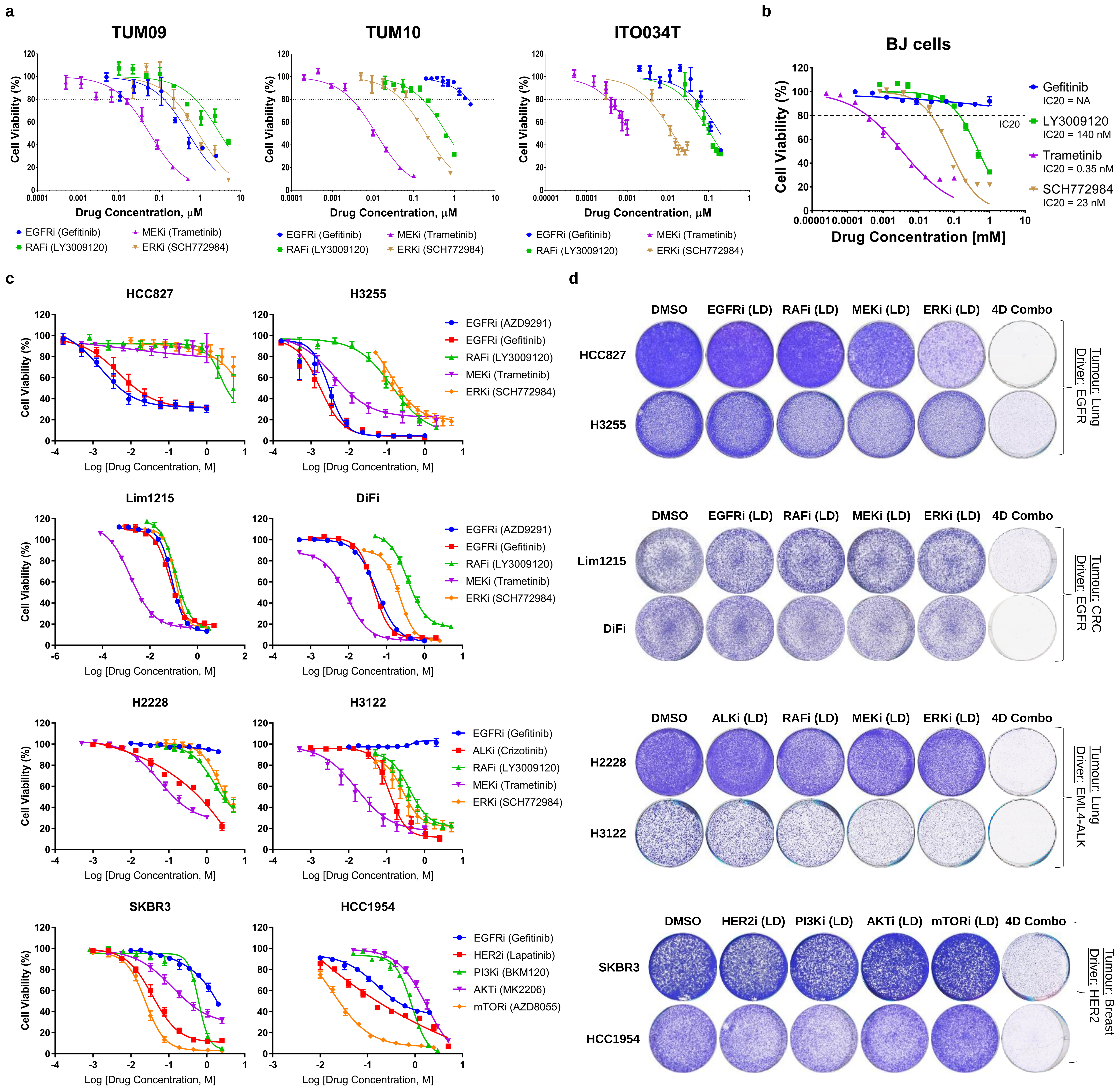

Supplementary Figure 3: MLD therapy is effective in multiple cancer cell lines.

a-c, Dose-response curves across the organoid and cell line panel. **a** ,Organoids were cultured with DMSO or with the different inhibitors and after 5 days of drug treatment cell viability was measured using CellTiter-Glo®. SEM from 3 replicates is plotted. **b**, **c**, Cells were plated in 384-well plates. Drugs were added ~24h after plating; after 4 days of exposure to the drugs cell viability was measured using CellTiter-Blue®. SEM from 3 replicates is plotted. Low doses (IC20s) were then determined (see Supplemental Table 1). **d,** MLD therapy is effective in several cell lines/tumour types. HCC827, H3255, Lim1215, DiFi, H2228, H3122, SKBR3 and HCC1954 cell lines were treated with DMSO, with the indicated pathway inhibitors at low dose and with their combination (4D Combo). After 10 days of treatment plates were stained and scanned.

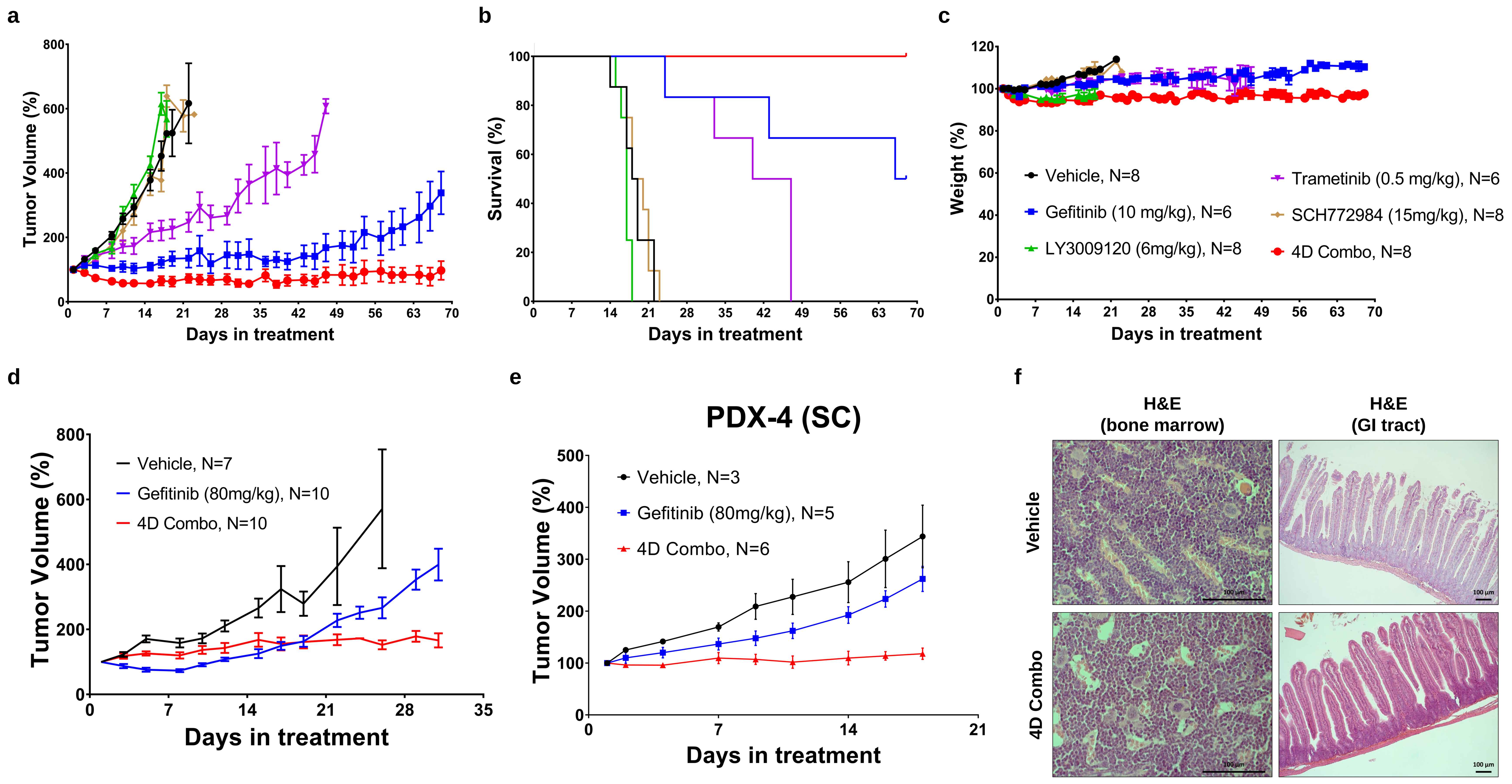

Supplementary Figure 4: MLD therapy reduces tumour volume *in vivo* without toxicity.
a-d, PC9 xenografts are sensitive to 4D Combo without toxicity. (**a-c**) PC9 cells were grown as tumour xenografts in BALB/cAnNRj-Foxn1nu mice. After tumour establishment (200–250 mm3), mice were treated 5 days/week with vehicle (N=8), gefitinib (10 mg/kg) (N=6), LY3009120 (6 mg/kg) (N=8), trametinib (0.5 mg/kg) (N=6), SCH772984 (15 mg/kg) (N=8) or the combination of the 4 inhibitors (4D Combo) (N=8) for 10 weeks. In **a** the mean tumour volume percentages ± SEM is shown; In **b** the Kaplan-Meier survival curve is shown and in **c** the mice weight percentages ± SEM is shown. **d**, PC9 cells and $PC9^{GR}$ cells were mixed in a 9:1 ratio, respectively, and were grown as tumour xenografts in BALB/cAnNRj-Foxn1nu mice. After tumour establishment (200–250 mm3), mice were treated 5 days/week with vehicle, with the MTD of gefitinib (80 mg/kg) and with 4D Combo – cocktail containing gefitinib (1 mg/kg), LY3009120 (6 mg/kg), trametinib (0.1 mg/kg), SCH772984 (15 mg/kg) for 30 days. The mean tumour volume percentages ± SEM is shown. **e,** EGFR and p53 mutant PDX responds to 4D Combo. PDX-4 was generated from a biopsy of patient with EGFR and TP53 mutation that progressed after afatinib and chemotherapy treatment. After tumour establishment, mice were treated 5 days/week with Vehicle (N=3), with gefitinib (80 mg/kg) (N=5) or with 4D combo (N=6) – cocktail containing gefitinib (10 mg/kg), LY3009120 (6 mg/kg), trametinib (0.5 mg/kg) and SCH772984 (15 mg/kg) (N=6) for 18 days. Tumour volume percentages ± SEM is shown. **f,** H&E stainings from the GI tract and the bone marrow of the PC9 xenografts in **a**. A representative staining image from the vehicle and 4D combo cohorts (N=8) is displayed. Scale bars 100µm.

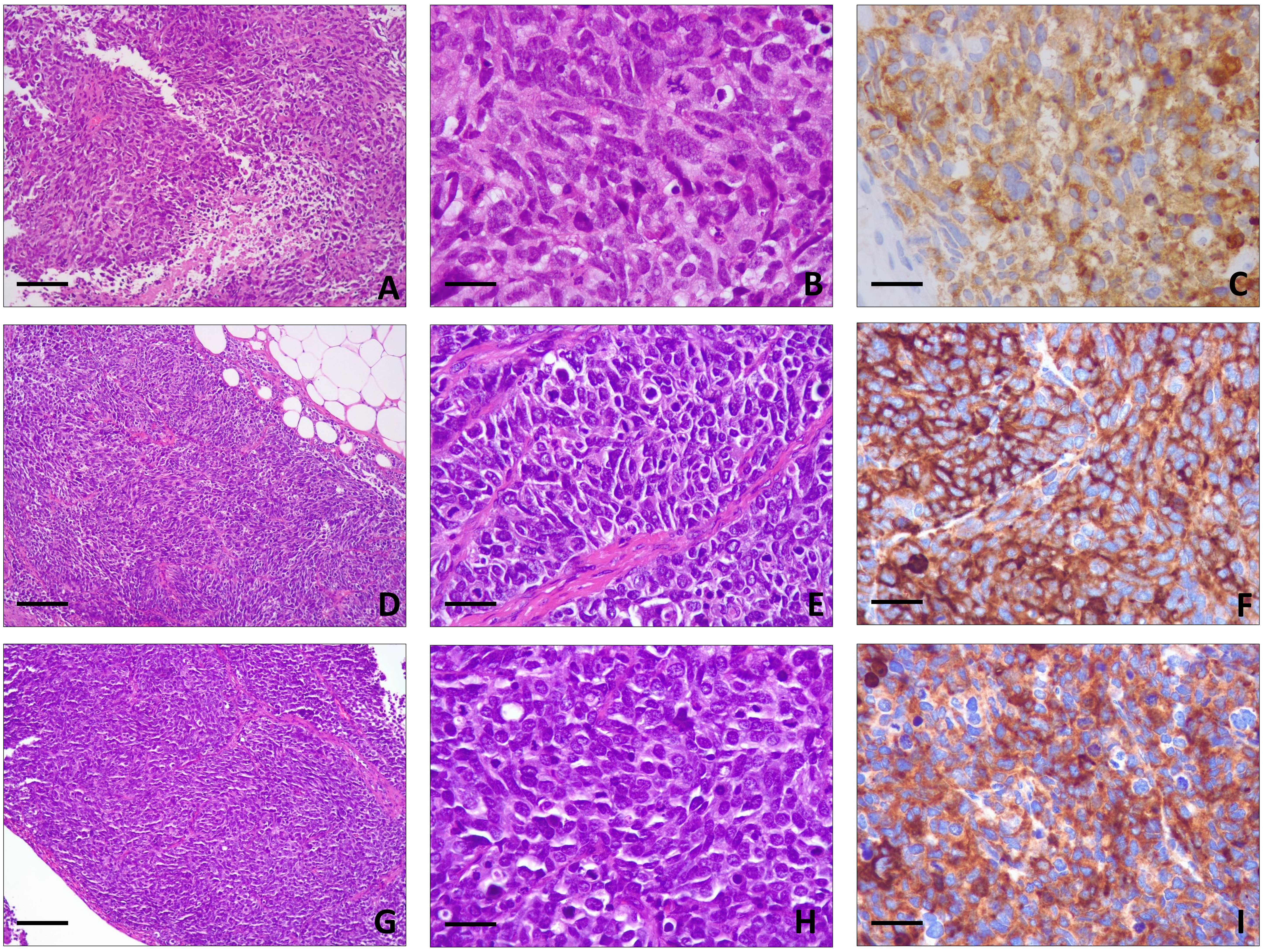

Supplementary Figure 5: Small cell lung cancer transformation of PDX4.
Biopsy samples from either the primary human lung cancer (**a-c**), the subcutaneous xenograft of this tumor (**d-f**) or the orthotopic xenograft of this tumor (**g-i**) were fixed and stained with H&E. Immunohistochemical staining with synaptophysin antibody was performed to assess small cell lung cancer transformation.
a-c: Small cell carcinoma metastatic to a mediastinal lymph node. The tumor has a sheet-like growth pattern. The tumor cells have ovoid or spindled nuclei and scant cytoplasm. Nuclear chromatin is finely granular and nucleoli are absent. There are brisk mitotic activity with atypical mitotic figures. **d-f**: subcutaneous xenograft of lung cancer shown in **a-c**. **g-i**: orthotopic xenograft of lung cancer shown in **a-c**. **a, d** and **g**: original magnification 100X, scale bars 200 µm. **b, c, e, f, h, i**: original magnification 400X, scale bars 50 µm. **a, b, d ,e, g, h**: H&E stain. **c, f, i**: immunohistochemical stain for synaptophysin. A representative staining image is displayed from the patient biopsy (**a-c**), from a total of 8 blocks from 8 different mice (**d-f**) and from 6 blocks from 6 different mice (**g-i**).

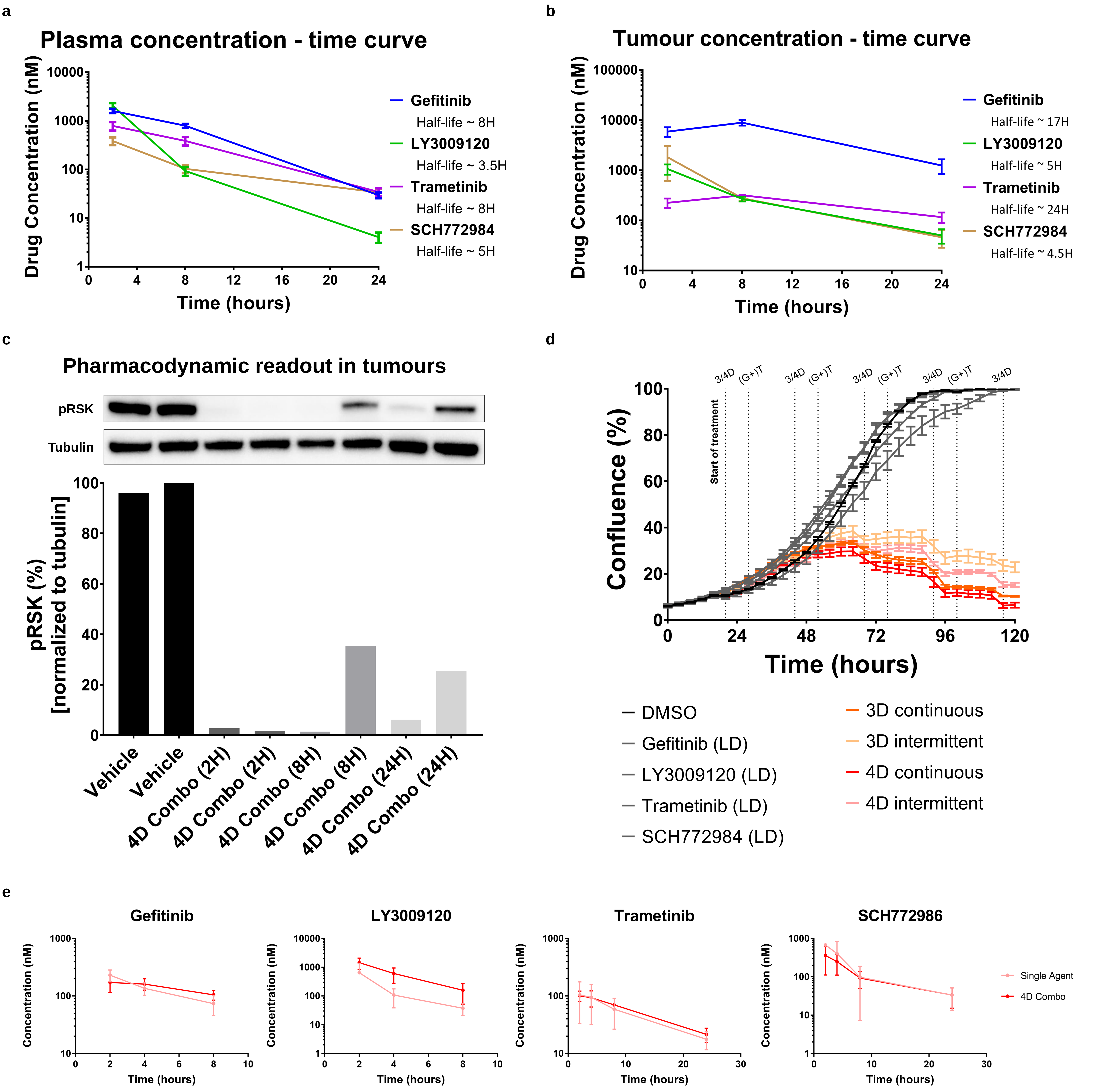

Supplementary Figure 6: PK-PD studies in PC9 xenografts reveal different half-lives of the inhibitors.
a-d, Pharmacokinetic and pharmacodynamics studies in PC9 xenografts. In **a-c** PC9 cells were injected (bilaterally) subcutaneously in BALB/cAnNRj-Foxn1nu mice. After tumour establishment (~200 mm3), mice were treated with vehicle (N=4) or 4D Combo (N=12). Vehicle mice were sacrificed 2H after treatment; Mice treated with 4D combo were sacrificed 2, 8 and 24h after treatment, respectively; 4 mice were sacrificed per time point. Blood and tumours were harvested; half of the tumour was used for the PD study and the other half was used for biochemical analysis. The drug concentrations in the blood and in the tumours were determined by mass spectrometry. In **a** the concentration of the individual drugs in the plasma is displayed and in **b** the concentration of the individual drugs in the tumours is displayed; SEM is plotted. In **c** the level of pathway inhibition in the tumours was measured by examining pRSK protein levels in the western blot (WB). Tubulin was used as loading control. WB was quantified using the Image Lab V5.2.1 software, from Bio Rad. **d,** Intermittent MLD therapy is less efficient in reducing cell growth in PC9 cells. PC9 cells were plated and incubated overnight to allow attachment to the plate. Cells were then treated with DMSO, with EGFR, RAF, MEK, ERK inhibitors at low dose, with 3D Combo or with 4D Combo. To mimic the availability of the drugs *in vivo*, in some of the 3D and 4D combo replicates the RAF and ERK inhibitors were removed from the culture media for approximately 8 hours every day (called intermittent MLD therapy). Confluence over time was measured by the IncuCyte®. SEM from 3 replicates is plotted. **e,** BALB/cAnNRj-Foxn1nu mice were treated with gefitinib (1mg/kg, N=3), LY3009120 (6mg/kg, N=3), trametinib (0,1mg/kg, N=3), SCH772984 (15mg/kg, N=3) or 4D Combo (N=3) and blood was harvested 2, 4, 8 and 24h after treatment. The drug concentrations in the blood was determined by mass spectrometry. The concentration of the drugs in the plasma given as single agent or in the 4D Combo ± SEM is displayed.

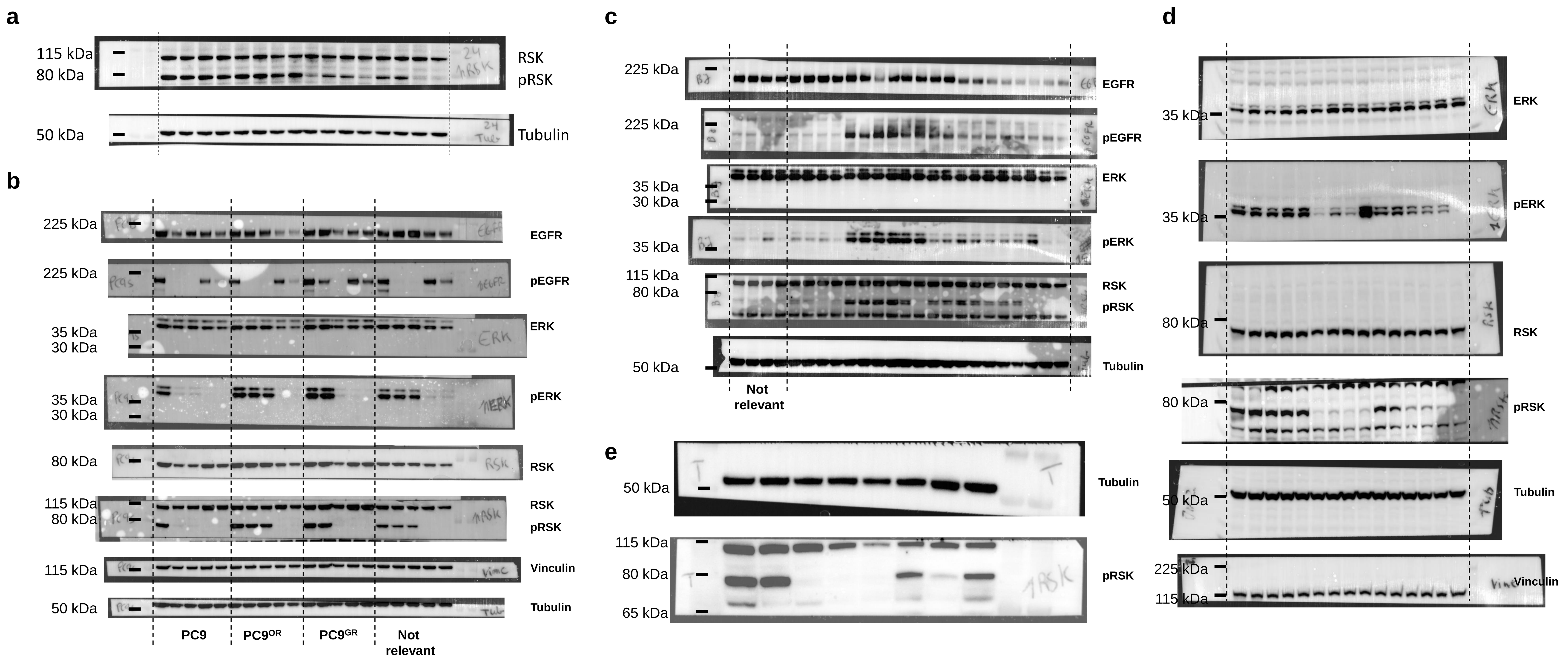

Supplementary Figure 7: Full blots images.

a, Figure 1e. **b,** Figure 2d. **c,** Figure 3c. **d,** Supplemental Figure 1b. **e,** Supplemental Figure 6c.

						Drug concentrations used in the study (µM)							
						Low Doses				High Doses			
**Cell Line Name**	**Tissue of Origin**	**Driver**	**Plating density (384-well plates)**	**Plating density (96-well plates)**	**Plating density (6-well plates)**	**EGFRi (Gefitinib)**	**RAFi (LY3009120)**	**MEKi (Trametinib)**	**ERKi (SCH772984)**	**EGFRi (Gefitinib)**	**RAFi (LY3009120)**	**MEKi (Trametinib)**	**ERKi (SCH772984)**
PC9/PC9^{OR}/PC9^{GR}	Lung	EGFR	150 cells	2000 cells	20000 cells	0.007	0.25	0.008	0.25	0.28	2.8	0.32	2.5
HCC827		EGFR	350 cells	NA	40000 cells	0.0025	0.5	0.1	0.75	1	5	1	5
H3255		EGFR	750 cells	NA	50000 cells	0.001	0.03	0.001	0.04	1	2	2	5
**Cell Line Name**	**Tissue of Origin**	**Driver**	**Plating density (384-well plates)**	**Plating density (96-well plates)**	**Plating density (6-well plates)**	**ALKi (Crizotinib)**	**RAFi (LY3009120)**	**MEKi (Trametinib)**	**ERKi (SCH772984)**	**ALKi (Crizotinib)**	**RAFi (LY3009120)**	**MEKi (Trametinib)**	**ERKi (SCH772984)**
H3122	Lung	EML4-ALK	1000 cells	NA	60000 cells	0.075	0.11	0.0038	0.084	2.5	5	1	5
H2228		EML4-ALK	1500 cells	NA	80000 cells	0.046	0.5	0.01	0.75	2.5	5	1	5
**Cell Line Name**	**Tissue of Origin**	**Driver**	**Plating density (384-well plates)**	**Plating density (96-well plates)**	**Plating density (6-well plates)**	**EGFRi (Gefitinib)**	**RAFi (LY3009120)**	**MEKi (Trametinib)**	**ERKi (SCH772984)**	**EGFRi (Gefitinib)**	**RAFi (LY3009120)**	**MEKi (Trametinib)**	**ERKi (SCH772984)**
DiFi	CRC	EGFR	1000 cells	NA	60000 cells	0.02	0.17	0.0025	0.095	2	5	0.5	2.5
Lim1215		EGFR	1250 cells	NA	40000 cells	0.04	0.06	0.0006	0.075	5	3	0.5	2.5
**Cell Line Name**	**Tissue of Origin**	**Driver**	**Plating density (384-well plates)**	**Plating density (96-well plates)**	**Plating density (6-well plates)**	**HER2i (Lapatinib)**	**PI3Ki (BKM120)**	**AKTi (MK2206)**	**mTORi (AZD8055)**	**HER2i (Lapatinib)**	**PI3Ki (BKM120)**	**AKTi (MK2206)**	**mTORi (AZD8055)**
SKBR3	Breast	HER2	2500 cells	NA	100000 cells	0.025	0.3	0.05	0.025	2.5	2.5	2.5	2.5
HCC1954		HER2	500 cells	NA	40000 cells	0.0125	0.4	0.5	0.025	5	3	5	2.5
**Organoid Name**	**Tissue of Origin**	**Driver**	**Plating density (384-well plates)**	**Plating density (96-well plates)**	**Plating density (6-well plates)**	**EGFRi (Gefitinib)**	**RAFi (LY3009120)**	**MEKi (Trametinib)**	**ERKi (SCH772984)**	**EGFRi (Gefitinib)**	**RAFi (LY3009120)**	**MEKi (Trametinib)**	**ERKi (SCH772984)**
ITO034T	Lung	KRAS	NA	NA	NA	0.065	0.035	0.0003	0.004	NA	NA	NA	NA
TUM09	CRC	MEK	NA	NA	NA	0.125	0.76	0.014	0.225	NA	NA	NA	NA
TUM10		KRAS	NA	NA	NA	2.2	0.16	0.003	0.05	NA	NA	NA	NA

Supplementary Table 1: Compendium of drivers, plating density, low doses and high doses of the cell lines and organoids used in the study.

$PC9^{OR}$						$PC9^{GR}$					
#	Gene	Mutation	#	Gene	Mutation	#	Gene	Mutation	#	Gene	Mutation
1	ABHD12	c.*489C>A	56	NCOA6	c.514+43T>G	1	ABCA10	c.2344G>A	56	NTRK2	c.917C>A
2	ADAM19	c.*1650C>T	57	NDNF	c.1399G>A	2	ACAD8	c.742T>G	57	OAS2	c.*744C>T
3	AFF2	c.48-9T>G	58	NEURL2	c.480G>A	3	ALK	c.4146G>C	58	OR2M2	c.366C>T
4	ANKLE2	c.9G>A	59	NF2	c.1331C>T	4	AMY1C	c.-37T>C	59	OR4D6	c.85G>A
5	ARAP1	c.264C>T	60	NRG1	c.1163_1164delGTinsAG	5	ARID3A	c.766+5G>C	60	PIEZO1	c.3582C>G
6	ARHGEF11	c.3068-30G>A	61	NWD1	c.891G>C	6	ARMCX3	c.307C>T	61	PPP1R17	c.449G>A
7	ASTN2	c.1878G>A	62	OLFM4	c.357+38G>C	7	BAAT	c.829C>T	62	PROSER2	c.51G>A
8	B4GALNT3	c.-48C>A	63	OR10A2	c.259C>G	8	BBX	c.1028A>G	63	PRPF40A	c.834_869dupTGCTGTTGTTGCAGCAGCAGCAGCGGCAGCAGCAGC
9	BPTF	c.5303+23G>T	64	OR1S2	c.483G>A	9	BCL11A	c.1175T>C	64	PSG7	c.430+40G>C
10	BTBD17	c.*106G>T	65	PCDHB16	c.416C>A	10	CANX	c.1023T>C	65	RARA	c.179-5998C>T
11	CALY	c.440C>T	66	PCDHB4	c.2124G>T	11	CASP7	c.682+24G>C	66	RN7SKP187	n.-1759C>G
12	CASP1	c.818T>G	67	PCED1B	c.115A>G	12	CCDC149	c.1418G>A	67	RP11-195L15.2	n.-468G>C
13	CDC34	c.588G>A	68	PCLO	c.4529A>G	13	CCL21	c.68-34C>A	68	RP11-44F14.1	n.412C>G
14	CDH12	c.361G>C	69	PDE10A	c.198T>C	14	CEACAM4	c.*13G>A	69	SCFD2	c.1962+46G>A
15	CEBPZ	c.1983G>A	70	PLEC	c.8311G>C	15	CENPO	c.207G>A	70	SCN11A	c.1472delA
16	CEP120	c.*1003-7A>T	71	PMCH	c.438A>G	16	CHRNA3	c.1392T>C	71	SLC17A7	c.-887A>G
17	CLTC	n.-1471G>T	72	POLA2	c.204+34A>G	17	CIC	c.4203T>G	72	SMG7	c.1828C>T
18	CORO7	c.2422C>T	73	POM121	c.2975C>T	18	CRYBG3	c.5554G>T	73	SUSD3	c.231delG
19	DNMT3A	c.*4A>G	74	PPP4R1	c.2032A>G	19	CYP26B1	c.1040C>G	74	TBXAS1	c.1326+5G>A
20	EIF2B1	c.-52G>C	75	PRKAR2A	c.262+5G>T	20	DCAF1	c.493G>T	75	TG	c.7710G>T
21	EPHA6	c.500T>C	76	RAB34	c.-464_-447delCGCGCCCCGGGCCGCTC	21	DDX59	c.1258A>C	76	THAP9	c.581-13T>C
22	F3	c.279C>A	77	RP1-170O19.17	n.-2249C>T	22	DEPDC5	c.*438C>T	77	THUMPD2	c.907G>A
23	FAM184B	c.2456G>A	78	RSPH6A	c.58A>G	23	DTHD1	c.825T>C	78	TINF2	c.225T>C
24	FCRL4	c.722delG	79	RTN1	c.1587G>A	24	EGFR	c.2369C>T	79	TM6SF2	c.1008C>T
25	FCRL4	c.721A>T	80	RUNX1T1	c.88+13146C>G	25	EMCN	c.415+1474C>A	80	TNS1	c.150delA
26	GLI3	c.3372C>T	81	RYR2	c.7552C>A	26	ENAM	c.2492A>C	81	TRIM48	c.604C>G
27	GPRIN3	c.1489G>C	82	SARS2	n.-2409G>A	27	FEZ1	c.*114T>A	82	TRIO	c.4189A>C
28	GSTK1	c.*12G>A	83	SLC2A10	c.1447G>A	28	GFM1	c.153C>A	83	TRIO	c.4191A>T
29	GSTM2	c.230T>C	84	SLIT3	c.2108A>G	29	GLI3	n.1334-26C>G	84	TRMT2B	c.1071G>A
30	GUCA1C	c.132G>C	85	SPAG17	c.2907G>A	30	GLI3	c.2681C>G	85	UNCX	c.348G>C
31	HYAL4	c.334C>T	86	SPTB	c.2890G>A	31	GNAS	c.842A>G	86	VEPH1	c.1034_1047delAGAGCAGAGACATC
32	IL4R	c.1318A>G	87	STIP1	c.666C>G	32	GRIK3	c.1828C>A	87	XRN2	c.623T>C
33	IRS4	c.63_65delGGC	88	TM9SF2	c.974C>A	33	GSTK1	c.*12G>A	88	ZIC2	c.364C>T
34	KSR1	c.54C>G	89	TMEM108	c.1532C>T	34	GSTM2	c.230T>C	89	ZNF467	c.754C>G
35	KSR1	n.-1100C>T	90	TNXB	n.-732T>C	35	JAG2	c.2467G>C	90	ZNF644	c.1058A>G
36	LFNG	c.108A>G	91	TONSL	c.1590G>T	36	JAK3	c.3060C>T			
37	LINC01006	n.-4236delT	92	TRIP12	c.3483C>G	37	KCNK13	c.-1564C>G			
38	LIX1L	c.784_787dupCATT	93	TSHR	c.615-5308A>G	38	KIAA1109	c.3324-495T>C			
39	LPAR4	c.279T>A	94	TUBGCP2	c.2402G>T	39	KIAA1109	c.6763-58G>A			
40	LRRK1	c.590C>T	95	UNC93B1	n.-3171G>C	40	KIF1B	c.4400C>T			
41	MADCAM1	c.790C>T	96	UTRN	c.8337G>A	41	KIFAP3	c.41C>G			
42	MADCAM1	c.800_801delAGinsCC	97	WDR6	c.1012G>C	42	KMT2C	c.10898C>G			
43	MCF2	c.-278C>A	98	YDJC	c.362C>T	43	LRP4	c.199+14C>A			
44	MED28	c.150+6A>G	99	ZBTB25	c.*1322G>A	44	LTBP3	c.1978+15G>T			
45	MKNK2	c.*1725T>G	100	ZFHX4	c.1295C>G	45	MAEA	n.-82A>G			
46	MON1B	c.901C>T	101	ZNF423	c.-165+3206C>A	46	MAGEL2	c.1623_1624insT			
47	MTRR	c.1850+4T>A	102	ZNF451	c.*879G>C	47	MAGEL2	c.1623G>A			
48	MTRR	c.1850+6G>C	103	ZNF467	c.740C>T	48	MIOS	c.2608G>T			
49	MTUS2	c.634C>A	104	ZNF469	c.7162A>T	49	MYH2	c.2388C>T			
50	MUC16	c.40783+19G>A	105	ZNF479	c.806A>G	50	MYOC	c.630G>T			
51	MYH13	c.1973A>G	106	ZNF568	c.1161G>A	51	NARS2	c.-2497delT			
52	MYO18A	c.1002G>A	107	ZNF586	c.987A>G	52	NCOA7	c.573-41C>G			
53	NCAM2	c.738-2A>G	108	ZNF665	c.1597C>T	53	NDUFS3	c.157G>C			
54	NCKAP5	c.874A>T	109	ZWINT	c.-1_1delGAinsTT	54	NEMP2	c.564A>T			
55	NCOA6	c.5893+48_5893+52delCCTAA				55	NSL1	c.191-453G>T			

Supplementary Table 2: Compendium of *de novo* mutations found in $PC9^{OR}$ and $PC9^{GR}$ by Exome Sequencing.
For the characters nomenclature please check http://varnomen.hgvs.org/recommendations/general/

	PDX name			
	PDX-1	PDX-2	PDX-3	PDX-4
**Age**	46	69	44	39
**Gender**	Female	Male	Female	Female
**Smoking Info**	Former	Smoker	Former	Never
**Diagnostic**	Lung Adenocarcinoma	Lung Adenocarcinoma	Lung Adenocarcinoma	Lung Adenocarcinoma
**EGFR mutation**	Exon 19 deletion	Exon 19 deletion	L858R	Exon 19 deletion
**Treatment 1 (T1)**	Erlotinib	Erlotinib	Erlotinib	Afatinib
**Alterations after T1**	T790M positive	MET amplification	MET overexpression	Unknown
**Treatment 2**	Osimertinib	Gefitinib + Capmatinib	Gefitinib + Capmatinib	CBDCA + Pemetrexed
**Treatment 3**	Osimertinib + Capmatinib	CDDP + Pemetrexed	Carboplatin + Gemcitabine + Nivolumab	CDDP + VP-16
**Source of PDX**	After progressing to 2 lines	After progressing to 3 lines	After progressing to >3 lines	After progressing to 2 lines
**Known alterations in the PDX**	EGFR (T790M) and KRAS G12C	EGFR (del19) and METamp	EGFR (L858R) and METamp	TP53 mutation and SCLC transformation

Supplementary Table 3: Compendium of patient, tumour, treatments and mutations information for all the PDXs used in the study.
